# Analysis of the management of the tenth Ebola virus disease outbreak in the Democratic Republic of Congo: developing a multidisciplinary response model to strengthen the healthcare system during disease outbreaks

**DOI:** 10.1186/s12992-021-00775-4

**Published:** 2021-10-18

**Authors:** Bives Mutume Vivalya, Okesina Akeem Ayodeji, Yves Tibamwenda Bafwa, Louis Kasereka Muyisa, Astride Lina Piripiri, Jean-Bosco Kahindo Mbeva

**Affiliations:** 1 Department of Psychiatry, Kampala International University Western campus, Bushenyi, Uganda; 2 Department of Internal Medicine, Masereka Referral General Hospital, North- Kivu, Democratic Republic of the Congo; 3 Department of Human Anatomy, Kampala International University Western Campus, Kampala, Uganda; 4 Department of Internal Medicine, University of Bunia, Ituri Bunia, Democratic Republic of the Congo; 5 Department of Pediatrics, Musienene Referral General Hospital, North-Kivu, Democratic Republic of the Congo; 6 Kinshasa School of Public Health, Faculty of Medicine, University of Kinshasa, Kinshasa, Democratic Republic of the Congo; 7 Department of Public Health, Official University of Ruwenzori, North-Kivu, Democratic Republic of the Congo

**Keywords:** Democratic Republic of Congo, Emergency response, Ebola disease outbreak

## Abstract

The declaration of any public health emergency in the Democratic Republic of Congo (DRC) is usually followed by the provision of technical and organizational support from international organizations, which build a parallel and short-time healthcare emergency response centered on preventing the extension of health emergencies across the countries and over the world. Previous Ebola virus disease (EVD) outbreaks have highlighted the need to reinforce the healthcare sector in different countries.

Based on the difficulty to implement the International Health Regulations (2005) to the local level of affected countries including the DRC, this paper proposes a multidisciplinary model based on the health zones through the strengthening of preparedness and response structures to public health emergencies vis-à-vis the existing weak health systems existing in DRC. A commitment to integrating the emergency response in the existing health system should work to reduce the tension that exists between local recruitment and its impact on the quality of daily healthcare in the region affected by EVD outbreak on one hand, and the involvement of international recruitment and its impact on the trust of the population on the emergency response on the other. This paper highlights the provision of a local healthcare workforce skilled to treat infectious diseases, the compulsory implementation of training programs focused on the emergency response in countries commonly affected by EVD outbreaks including the DRC. These innovations should reduce the burden of health problems prior to and in the aftermath of any public health emergency in DRC hence increasing the wellbeing of the community, especially the vulnerable people as well as the availability of trained healthcare providers able to early recognize and treat EVD.

## Introduction

The declaration of any public health emergency is followed by the provision of technical support from international organizations to individual countries to limit the widespread of infectious diseases [1, 2]. As a result, an organized and short-time healthcare system to strengthen a healthcare workforce is built by international organizations based on the International Health Regulations 2005 (IHR 2005) [3]. The chain of the current emergency responses against the Ebola disease virus (EVD) outbreaks used an approach centered on the expert model, instead of its related weakness reported during the previous outbreaks [4, 5].

EVD outbreak is associated with important implications in concerning countries such as lack of trust in the government, food insecurity, loss of domestic income, stigmatization, impaired provision of healthcare, and disruption of school activities [6]. During EVD outbreaks, the full attention of national and international organizations usually focuses on the health emergency by steering the entire health sector [3]. In the Democratic Republic of Congo (DRC), the tenth EVD outbreak which occurred in a war-tone region took nearly two years and was characterized by 3470 reported cases and 2287 deaths, although it was marked by the use of approved drugs and vaccines against the Ebola virus by the United States Food and Drug Administration [7]. The response to the ongoing COVID-19 pandemic has highlighted the integration of the public health emergency response into the existing healthsystem [8]. Therefore, there is a need for building a model.

While conducting the analysis and reviewing the challenges identified during the tenth EVD outbreak in DRC, this paper proposes an alternative model based on a health system able to respond to future outbreaks that involve community engagement, the provision of skilled healthcare workers, and perform the quality of healthcare during outbreaks. Similar to the EVD outbreaks in the West-African region which caused 11,310 deaths and produced more than 17,300 orphans, the tenth EVD outbreak in DRC was marked by the declaration delay of a public health emergency, the contact tracing in an insecure setting, the lack of funds, the overwhelmed healthsystems, and the disruption of preventive interventions including immunization, HIV/AIDS, and tuberculosis prevention [9].

### Healthcare system in the Democratic Republic of Congo

**Fig. 1:**
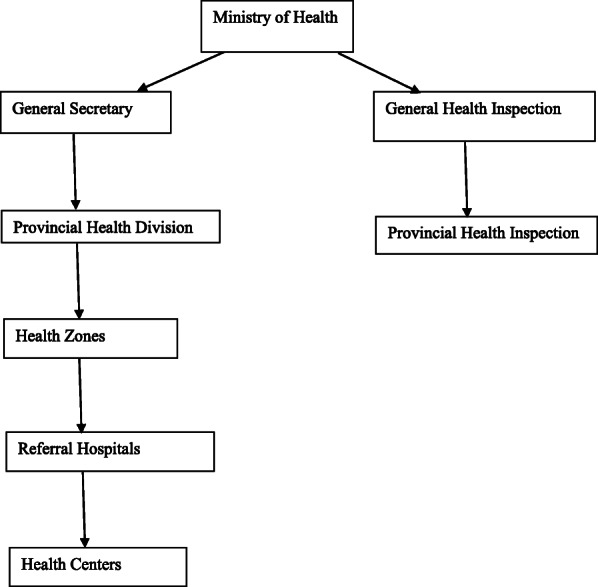
Congolese Health System Organization

The Health System in the DRC is based on three levels namely the central, intermediate, and operational levels (Fig. 1). The implementation level called operational level comprises 516 health zones built by community health centers, Reference health centers, and Referral Hospitals. This level is managed by a health committee extended to non-healthcare providers and led by the chairman of the health zone. The intermediate level which is responsible for technical and logistic support to health zones, links the implementation level to the central level of the Ministry of Health, and is managed by the provincial health division. The third level comprises the central level of the Ministry of Health with its cabinet and secretaries and has a normative role. The health sector in Congo is mainly controlled by the government, in collaboration with many Non-Governmental Organizations (NGOs) [10].

The DRC healthcare system is affected by the impoverishment of nearly 70 % of the population including health workers; poor adoption of the Health System Strengthening Strategy adopted in 2005, unequipped infrastructure, impaired supply of drugs into health facilities, multiples labor strikes motivated by poor salary, as well as the paucity of trained health workers [11]. This health system is supported by the WHO and numerous organizations including World Bank and European Union [8]. Community engagement based on cultural considerations, the involvement of non-health stakeholders, the existence of health committees in health areas are some of the advantages of the DRC health system [10]. Despite the weakness of the health system in DRC, the North Kivu is marked by a health system built on the involvement of community mobilization, the availability of trained health workers, and a drug store for the distribution of medicine within the health zones, and close and continuous support from numerous organizations. This system was the core of the management of the twelfth EVD outbreak which occurred in Butembo city on March 1, 2021; and which took less than three months However, health workers of all health zones in North-Kivu and Ituri provinces are not skilled to recognize and respond to outbreaks [10]. Most local healthcare workers had left their daily activities in different health facilities then they were recruited into different commissions of the emergency response team due to the financial benefit commonly reported during the outbreaks.

### Analysis and limitations revealed during the management of EVD in DRC

The history of emergency response to outbreaks has been linked to the early attempts to implement a commitment focused on anticipatory actions and the performance of the health system [3]. In the second part of the twentieth century concerned by enormous barriers to health delivery services during public health emergencies [12], international organizations including the World Health Organization (WHO) established the health emergency programs as a reform built to improve the health system and to reduce health threats among affected communities [13]. In DRC, the emergency response is fully performed and supported by NGOs which not only installed a parallel health system but also supported the existing health system via technical, financial, and logistical means to health facilities. Establishing with a purpose of shortening the EVD outbreak burden, the emergency response against the EVD outbreaks faced numerous challenges due to the lack of emergency response work plans regarding the EVD management including the shortage of trained healthcare workers, impaired health communication, the lack of functional laboratory networks, poor community engagement systems, and the overwhelming of existing health system prior to the EVD [5]. These challenges were highlighted during the tenth EVD outbreak which occurred in a DRC setting characterized by armed conflict, high density, and mobility of communities [14].

Instead of recruiting the local healthcare workers as the emergency responders in an outbreak situation, international response staff was used during the tenth EVD outbreak. The massive participation of international staff workers involved in the emergency response team and their increased salary compared to local health workers lead to increased community resistance illustrated by the burns of Ebola treatment facilities and the murder of Richard Valery Mouzoko, a WHO staff on April 19, 2019, in Butembo city, located in North-Kivu province [15]. Although during that period healthcare services were free of charge in public health facilities as a response to the outbreak, health facilities are marked by poor utilization by patients due to excessive fear of potentially being referred to the Ebola treatment center. Additionally, most of these facilities did not have enough health workers given that their engagement and involvement in the outbreak response [6]. The impairment of the quality of care, the increased frustration of local health workers, and the worsening of numerous health indicators were reported [9] to impair the weak health system of DRC [16]. In view of these multiple challenges, there is a need for a revision of the implementation of the IHR policies [3] at the scale of DRC.

### Basis of the health system reforms to manage EVD in war-zone settings: case of North-Kivu and Ituri Provinces

A functional health system focused on the preparedness and response services in health emergencies [17] should be created to promote an emergency response based on the existing health system in DRC [18]. The overall incidence of 5 % Ebola virus infections among healthcare workers during the tenth EVD outbreak demonstrated that infection, prevention and control measures are not fully applied at health facilities of health zones in the North-Kivu and Ituri provinces.

The tenth EVD outbreak has constituted a clear opportunity to build the capacity of a strong health system in DRC via the training of a local healthcare task force [13], and the involvement of community practitioners in different commissions to handle any health problems during outbreaks [5]. Therefore, a new organizational model based on the enhancement of a health communication system, and the development of an effective monitoring system to improve the capacity of existing provincial and district health systems in DRC was suggested by a recent study carried out by Mbeva and colleagues [19]. Fortnightly meetings centered on preparedness, prevention [20]; and response against any public health problems should be organized at an intermediary level in addition to regular supervision of the local health force, centered on the utilization of services and resources planning [3]. The shift of basic formation of healthcare workers with a particular focus on preventing and treating infectious diseases with a high risk of outbreaks should be encouraged by the educational and health ministries. A multidisciplinary team integrated into the existing health system is needed to provide equitable access to health care during outbreaks. A well-organized health system between international organizations and the local health system should allow adequate supervision and continuous medical education based on common health problems [21].

### Proposals to improve the health system in public health emergency settings

**Fig. 2:**
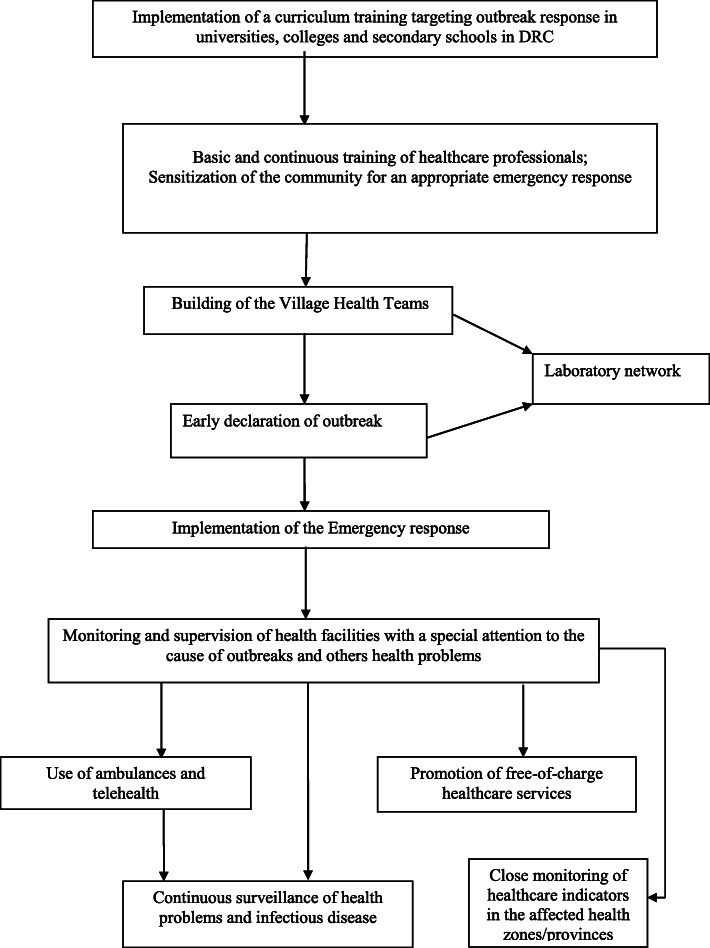
Proposals to improve the Health System in Public Health Emergency Settings

The management of the tenth EVD outbreak in DRC has tinted the reinforcements of the health systems by enhancing primary and specialized healthcare capacities either during a global crisis or in normal conditions. This sustained approach (Fig. 2) may provide an opportunity to set up mitigation strategies for universal health coverage and raise the awareness of severe emerging diseases with community engagement, the provision of facilities and funds [22], and a better-equipped infrastructure [23].

First, the emergency response against EVD outbreaks is usually impaired by community resistance. However, the experience of global health security in the previous public health problems showed that community resistance is encouraged by the non-recruitment of local practitioners and the lack of funds [24]. In Uganda, the response against the EVD outbreak showed that an organized health system involving the community at the grass-root level known as the Village Health Teams allowed the early recognition of outbreaks through the daily surveillance meetings and contact tracing [25]. Thus, close communication between the community and health facilities might be implemented during or not the health emergency. Community volunteer health workers should be continuously skilled to recognize the cause of outbreaks [25].

Secondly, the lack of local healthcare workers trained to manage a public health emergency in North-Kivu and Ituri has affected the EVD outbreak, called for international healthcare workers, and increased community resistance [3]. Building the healthcare workforce capacity able to handle the outbreaks requires a continuous provision of a multidisciplinary team able to participle in the public health emergencies response team via a specialized training plan centered on the early recognition of symptoms of pandemics and outbreaks [8]. Given that trained health worker has an increasing work-trade; financial motivation and training of all stakeholders have to be addressed by the government of the DRC and to ensure the retention of talented and well-trained staff needed to respond against EVD outbreak in their respective positions. Additionally, a work plan of the Ministry of Health should emphasize the provision of equipped facilities and funds for essential medicine, vaccines, laboratories, and personal protective types of equipment. Furthermore, a strong laboratory network of all health zones has to be implemented to allow enabling timely diagnosis and management of outbreaks [26].

Thirdly, monitoring, contact tracing, and supervision have been criticized over the years in their methods of handling infectious disease outbreaks. However, the close monitoring and the recruitment of local healthcare workers and community leaders have shown a positive result during the response against the tenth EVD outbreak. Also, trained staff should be encouraged to conduct continuous monitoring and supervision of health facilities with special attention to the cause of outbreaks. Given that the communication is impaired between the health facilities of different levels of the ministry of health and the community, the use of available means of communication such as community radios and community outreach programs could promote awareness of health problems and quick health communication. The extension of the job description of community health workers to address public health challenges and to support the appropriate management of many diseases at the community level should be supported [27].

The lack of ambulances and means of communication especially in rural health zones impaired the chain of early treatment from the community, primary health facilities, and the Ebola treatment center.

The occurrence of EVD outbreaks in DRC, commonly in rural and armed conflict settings, demonstrated the role of delayed consultation of health facilities by patients in the early recognition of outbreaks. This delay is caused by the lack of money for the affected patients. Therefore, the promotion of a free-of-charge healthcare service should be promoted up in health zones with a high risk of outbreaks, during or out of global health crisis.

### The integrative proposed model

This paper proposes a multidisciplinary model made to increase the health system capacity in regions concerned with public health emergencies including DRC, especially in North Kivu and Ituri provinces. This model will be applied by the Ministry of Health and other related agencies involved in public health emergencies including WHO, especially in armed conflict and developing settings. This model is based on two main assumptions: firstly, the model centered on the IHR (2005) may increase the community resistance; modulates the worseness of the health system during the outbreaks, and impaired the management of common health problems [5]. The second postulates is that the integration of emergency response into the health system may not only allow to reduce the economic, health, and social impacts related to the outbreaks but also will help healthcare workers and health facilities to be able to handle the next outbreaks without the recruitment of international healthcare workers.

**Fig. 3:**
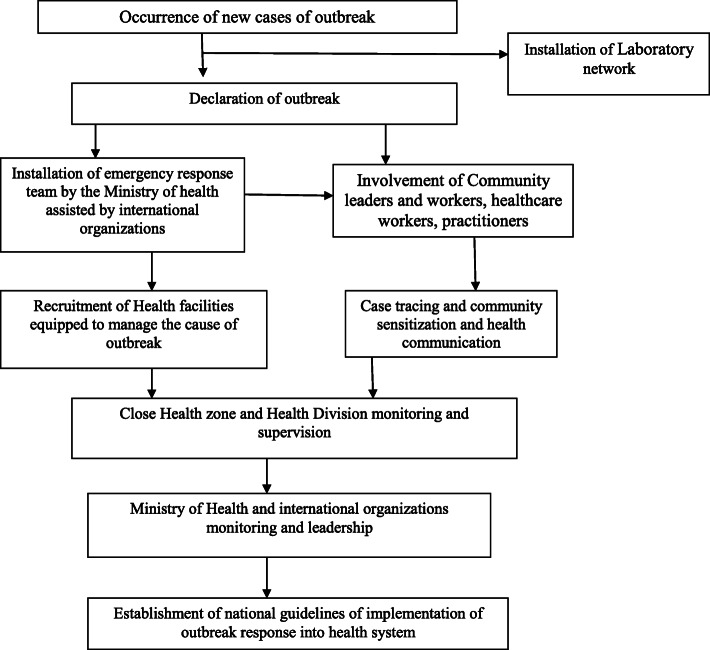
The integrative health system proposed during EVD outbreaks

The integrative model may reduce the delay of outbreak recognition and modulates the early contact tracing and contention of an outbreak in an efficient way. Therefore, the shift from a model based on a short-term parallel health system into an emergency response model integrated into the existing health systems could accelerate the elaboration of this model [8]. Given that this model may be seen as a threat to both individuals and organizations that used to benefit from the parallel health emergency response used to fight EVD outbreaks, this model emphasizes the integration of emergency response into the existing health system. Therefore, it may strengthen the health system in DRC by building the capacity for early recognition and treatment of EVD. This approach will be constituted by workers from peripheral to central levels, trained and skilled to respond to the health emergency response. This process may encourage the recruitment of a workforce with background knowledge of emergency response and it will be used as the focal point for the reinforcement of health systems. Therefore, the community and health workers will have the privilege to be continuously trained for the early diagnosis of public health problems. Subsequently, a health emergency response program could be integrated into the course content of medical and allied health students, to get emergency response skills, depending on the existing and available means. This model will be carried out by the health workers in their specific health facilities, as it is currently done during the COVID-19 in DRC. A criticism of this model could be the occurrence of the initial outbreak in an area that has not been concerned by health emergencies previously. The prevention of infectious disease could take account of the epidemiological settings with the help from the veterinary, farmer, and other non-health stakeholders [8] (Fig. 3).

## Conclusions

The tenth EVD outbreak in DRC has revealed the need for new approaches to strengthen the weak health systems in developing countries. The lessons from previous outbreaks have emphasized the integration of the emergency response into the existing health system. There is a need to set up the reinforcement of the operational health level to perform the readiness and preparedness against any public health emergency. Therefore, the multidisciplinary model centered on the health zone is proposed to fight infectious diseases that cause outbreaks. The trained health workers on providing emergency health care services are required as well as support from international organizations for effective management of health emergencies and disease outbreaks the first time. Finally, a monitoring system by the central and intermediary level of the ministry of health in these countries must be instituted, which should be supported and supervised by the WHO.

## Data Availability

Not applicable.

## Abbreviations

CDC: Centers for Disease Control and Prevention -always with s as there are many of them
COVID-19: Corona Virus Disease-19
DRC: Democratic Republic of Congo
EVD: Ebola disease outbreaks
IHR: International Health Regulations
NGO: Non-Governmental organization
WHO: World Health Organization
